# Supplementary material for: Quality of Life in a Cohort of 1078 Women Diagnosed with Breast Cancer in Spain: 7-Year Follow-Up Results in the MCC-Spain Study
Source: Int J Environ Res Public Health. 2020 Nov 13;17(22):8411. doi: 10.3390/ijerph17228411 (PMC7696097; doi:10.3390/ijerph17228411)
Supplement: Supplementary file 1 [file ijerph-17-08411-s001.pdf]

Title: Quality of life in a cohort of 1078 women diagnosed with breast cancer in Spain: 7-year follow-up results in the MCC-Spain study.

Journal: Quality of Life Research

Authors: Jéssica Alonso-Molero*, Trinidad Dierssen-Sotos, Inés Gómez-Acebo, Nerea Fernández de Larrea Baz, Marcela Guevara, Pilar Amiano, Gemma Castaño-Vinyals, Tania Fernández-Villa, Victor Moreno, Juan Bayo, Ana Molina-Barcelóa, María Fernández-Ortiz, Claudia Suarez-Calleja, Rafael Marcos-Gragera, Xavier Castells, Gil-Majuelo Leire, Eva Ardanaz, Beatriz Pérez-Gómez, Manolis Kogevinas, Marina Pollán and Javier Llorca.

*Corresponding author: University of Cantabria – IDIVAL, Santander, Spain. alonsomoleroj@gmail.com

Table S1. Main characteristics of the followed patients.

Variable	Category	Breast cancer (n = 1685)
Age (mean±sd)		56.5 (±12.6)
Gender	Women	1685 (100%)
Postmenopausal	Yes	1095 (65.0%)
	No	589 (35.0%)
	Missing	1 (0.1%)
Histology (specific types in each tumour)		Ductal: 1276 (75.7%)
		Lobular: 110 (6.5%)
		Paget disease: 19 (1.1%)
		Others: 280 (16.6%)
Tumour size	T0	23 (1.4%)
	T1	861 (51.1%)
	T2	424 (25.2%)
	T3	73 (4.3%)
	T4	39 (2.3%)
	Tis	109 (6.5%)
	Missing	156 (9.3%)
Node infiltration	N0	877 (52.0%)
	N1	441 (26.2%)
	N2	186 (11.0%)
	N3	5 (0.3%)
	Missing	176 (10.4%)
Metastasis	No	1376 (81.7%)
	Yes	41 (2.4%)
	Missing	268 (15.9%)
	I	702 (41.7%)
	II	479 (28.4%)
	III	179 (10.6%)
	IV	22 (1.3%)
	Missing	303 (18.0%)

Table S2. Description of the sociodemographic variables at diagnosis according to answering or not QoL questionnaires.

		QoL questionnaire (N(%))				
		Answered	Refused to answer	No located	Deceased	p-value
Age at diagnosis	mean ± sd	54.86(11.31)	59.63(13.48)	55.61(13.56)	61.54(15.32)	<0.001
Menopausal status	Postmenopausal	637(59.09)	53(66.25)	173(53.89)	143(69.42)	0.12
	Premenopausal	441(40.91)	27(33.75)	148(46.11)	63(30.58)	
Family history of breast cancer	None	786(72.91)	58(72.50)	248(77.26)	162(78.64)	0.01
	First degree	156(14.47)	14(17.50)	51(15.89)	27(13.11)	
	Second degree	127(11.78)	7(8.75)	21(6.54)	14(6.80)	
	Missing	9(0.83)	1(1.25)	1(0.31)	3(1.46)	
Educational level	Less than primary	142(13.17)	16(20.00)	56(17.45)	48(23.30)	<0.001
	Primary education	351(32.56)	25(31.25)	104(32.40)	75(36.41)	
	Secondary education	368(34.14)	26(32.50)	106(33.02)	53(25.73)	
	University	217(20.13)	13(16.25)	55(17.13)	30(14.56)	
Civil status	Single	131(12.15)	11(13.75)	44(13.71)	35(16.99)	<0.001
	Married	750(69.57)	44(55.00)	212(66.04)	115(55.83)	

	Live in company	90(8.35)	14(17.50)	26(8.10)	11(5.34)	
	Widow	105(9.74)	11(13.75)	36(11.21)	44(21.36)	
	Missing	2(0.19)	0(0.00)	3(0.93)	1(0.49)	
Smoking	No smoker at diagnosis	587(54.45)	46(57.50)	169(52.65)	143(69.42)	0.01
	Former smoker at diagnosis	295(27.37)	19(23.75)	80(24.92)	37(17.96)	
	Smoker at diagnosis	196(18.18)	15(18.75)	72(22.43)	26(12.62)	
BMI	<18.5	23(2.13)	3(3.75)	1(0.31)	3(1.46)	<0.001
	18.5-24.9	508(47.12)	26(32.50)	155(48.29)	74(35.92)	
	25-29.9	355(32.93)	33(41.25)	100(31.15)	84(40.78)	
	≥30	192(17.81)	18(22.50)	65(20.25)	45(21.84)	

Table S3. Description of tumour characteristics according to answering or not QoL questionnaries.

			**QoL questionnaire (N(%))**				**p-value**
			**Refused to answer**	**Answered**	**No located**	**Exitus**	
**Tumour characteristics**	Tumor size	T0	0(0.00)	17(1.58)	2(0.62)	4(1.94)	<0.001
		T1	41(51.25)	611(56.68)	166(51.71)	58(28.16)	
		T2	23(28.75)	248(23.01)	79(24.61)	74(35.92)	
		T3	3(3.75)	36(3.34)	12(3.74)	22(10.68)	
		T4	3(3.75)	12(1.11)	6(1.87)	18(8.74)	
		Tis	4(5.00)	87(8.07)	15(4.67)	3(1.46)	
		Missing	6(7.50)	67(6.22)	41(12.77)	27(13.11)	
	Node infiltration	N0	39(48.75)	625(57.98)	153(47.66)	60(29.13)	<0.001
		N1	19(23.75)	286(26.53)	80(24.92)	56(27.18)	
		N2	13(16.25)	67(6.22)	28(8.72)	34(16.50)	
		N3	3(3.75)	17(1.58)	11(3.43)	18(8.74)	
		Missing	6(7.50)	83(7.70)	49(15.26)	38(18.45)	
	Metastasis	No	67(83.75)	930(86.27)	254(79.13)	132(64.08)	
		Yes	3(3.75)	5(0.46)	5(1.56)	29(14.08)	<0.001
		Missing	10(12.50)	143(13.27)	62(19.31)	45(21.84)	
	Complete clinical remission	No	2(2.50)	24(2.23)	9(2.80)	42(20.39)	<0.001
		Yes	74(92.50)	964(89.42)	298(92.83)	150(72.82)	
		Missing	4(5.00)	90(8.35)	14(4.36)	14(6.80)	
	Recurrence	No	71(88.75)	927(85.99)	268(83.49)	54(26.21)	<0.001
		Yes	3(3.75)	37(3.43)	30(9.35)	96(46.60)	
		Missing	6(7.50)	114(10.58)	23(7.17)	56(27.18)	
	TNM pathological stage	I	32(40.00)	515(47.77)	123(38.32)	32(15.53)	<0.001
		II	21(26.25)	320(29.68)	85(26.48)	53(25.73)	
		III	15(18.75)	90(8.35)	35(10.90)	38(18.45)	
		IV	3(3.75)	5(0.46)	5(1.56)	29(14.08)	
		Missing	9(11.25)	148(13.73)	73(22.74)	54(26.21)	
	Histological grade	Well differentiated	17(21.25)	228(21.15)	66(20.56)	18(8.74)	<0.001
		Moderately differentiated	19(23.75)	328(30.43)	118(36.76)	55(26.70)	
		Poorly differentiated	18(22.50)	209(19.39)	70(21.81)	58(28.16)	
		Missing	26(32.50)	313(29.04)	67(20.87)	75(36.41)	

Intrinsic subtype	Luminal A	54(67.50)	664(61.60)	181(56.39)	98(47.57)	<0.001
	Luminal B	14(17.50)	196(18.18)	75(23.36)	46(22.33)	
	Her2	3(3.75)	56(5.19)	5(1.56)	17(8.25)	
	Basal-like	3(3.75)	64(5.94)	24(7.48)	39(18.93)	
	Luminal ONI	4(5.00)	60(5.57)	25(7.79)	2(0.97)	
	Non-luminal ONI	1(1.25)	10(0.93)	2(0.62)	0(0.00)	
	Missing	1(1.25)	28(2.60)	9(2.80)	4(1.94)	

Luminal ONI: Luminal Otherwise Non Indicated (hormone receptors positive, HER2 receptors missing). Non-luminal ONI: Non-luminal Otherwise Non Indicated (hormone receptors negative, HER2 receptors missing.)

Table S4. Description of the treatment received.

			Questionnaire QoL (N(%))				p-value
			Refused to answer	Answered	No located	Exitus	
Treatment received	Immunotherapy	No	50(94.34)	760(91.02)	187(89.90)	75(88.24)	0.64
		Yes	3(5.66)	75(8.98)	21(10.10)	10(11.76)	
	Hormone therapy	No	17(32.08)	273(32.69)	86(41.35)	36(42.35)	0.05
		Yes	36(67.92)	562(67.31)	122(58.65)	49(57.65)	
	Chemotherapy	No	29(54.72)	425(50.90)	109(52.40)	44(51.76)	0.94
		Yes	24(45.28)	410(49.10)	99(47.60)	41(48.24)	
	HER2-targeted therapy	No	47(88.68)	698(83.59)	173(83.17)	77(90.59)	0.34
		Yes	3(5.66)	67(8.02)	16(7.69)	7(8.24)	
		Missing	3(5.66)	70(8.38)	19(9.13)	1(1.18)	
	Radiotherapy	No	8(15.09)	151(18.08)	53(25.48)	26(30.59)	<0.01
		Yes	43(81.13)	646(77.37)	125(60.10)	53(62.35)	
		Missing	2(3.77)	38(4.55)	30(14.42)	6(7.06)	
	Surgery	Conservative	44(83.02)	659(78.92)	153(73.56)	61(71.76)	0.15
		Mastectomy	9(16.98)	176(21.08)	55(26.44)	24(28.24)	

Based on women with TNM stage I or II

Table S5. Comparison between the means of PCS-12 and Monteagudo's CSF references values for each group of ages.

		This article PCS-12		Monteagudo's CSF Healthy and chronically disease women[20]		Monteagudo's CSF Healthy women[20]	
		n	Mean (95% CI) *	n	Mean (95% CI) **	n	Mean (95% CI) **
Age at diagnosis	35 – 44	163	47.30 (45.25, 49.36)	330	51.5 (50.67, 52.33)	149	53.5 (52.63, 54.37)
	45 – 54	334	45.20 (43.71, 46.68)	247	49.8 (48.6, 51.0)	78	54.2 (53.45, 54.95)
	55 – 64	329	45.34 (43.86, 46.83)	187	42.9 (41.02, 44.78)	19	53.5 (52.2, 54.8)
	65 – 74	164	44.07 (41.96, 46.17)	165	39.8 (37.89, 41.71)	12	49.6 (45.02, 54.18)
	≥75	56	40.57 (36.78, 44.36)	161	34.3 (32.31, 36.29)	7	47.3 (41.67, 52.93)

*Results adjusted for educational level, province of recruitment, stage breast cancer at diagnosis, and grade of differentiation. **Monteagudo's confidence intervals had been calculated for this article.
